# Diagnosis to Follow-Up: Practice Variability and Evidence-Based Gaps In Vital Pulp Therapy Among Saudi Dentists

**DOI:** 10.3290/j.ohpd.c_2444

**Published:** 2025-12-19

**Authors:** Yasir Dilshad Siddiqui, Osama S. Alothmani, Amna Yusuf Siddiqui, Ibrahem T. Almaktoom, Asrar Helal F. Alanazi, Khalid Maziad Alzabni, Haifa Ali Almutairi, Hmoud Ali Algarni, Farooq Ahmad Chaudhary

**Affiliations:** a Yasir Dilshad Siddiqui Assistant Professor, Department of Preventive Dentistry, College of Dentistry, Jouf University, Sakaka, Saudi Arabia. Conceptualisation, statistical analysis, and writing the manuscript.; b Osama S. Alothmani Assistant Professor, Department of Endodontics, Faculty of Dentistry, King Abdulaziz University, Jeddah, Saudi Arabia. Contributed substantially to the discussion, data collection, analysis, and proofreading of the manuscript.; c Amna Yusuf Siddiqui Assistant Professor, Department of Endodontics, Faculty of Dentistry, King Abdulaziz University, Jeddah, Saudi Arabia. Contributed substantially to data collection and proofread the manuscript.; d Ibrahem T. Almaktoom Researcher, College of Dentistry, Jouf University, Sakaka, Saudi Arabia. Contributed substantially to data collection, statistical analysis and proofreading the manuscript.; e Asrar Helal F. Alanazi Researcher, College of Dentistry, Jouf University, Sakaka, Saudi Arabia. Contributed substantially to the statistical analysis, writing the results section and proofreading the manuscript.; f Khalid Maziad Alzabni Researcher, College of Dentistry, Jouf University, Sakaka, Saudi Arabia. Contributed substantially to data collection, discussion and proofreading the manuscript.; g Haifa Ali Almutairi Researcher, College of Dentistry, Jouf University, Sakaka, Saudi Arabia. Contributed substantially to data collection and analysis, discussion and proofreading of the manuscript.; h Hmoud Ali Algarni Assistant Professor, Department of Restorative Dentistry, College of Dentistry, Jouf University, Sakaka, Saudi Arabia. Supervision, advisor, statistical analysis and proofreading the manuscript.; i Farooq Ahmad Chaudhary Associate Professor, School of Dentistry, Shaheed Zulfiqar Ali Bhutto Medical University, Islamabad, Pakistan. Conceptualisation, supervision, manuscript write-up, data analysis and proofreading the manuscript.

**Keywords:** bioactive materials, clinical practices, dental diagnostic tools, standardised treatment protocols, vital pulp therapy

## Abstract

**Purpose:**

Vital pulp therapy (VPT) is a minimally invasive approach aimed at preserving pulp vitality in cases of caries or trauma. Despite advancements in diagnostic tools and bioactive materials, clinical practices vary significantly. This study explored the preoperative, intraoperative, and postoperative practices of dental professionals in Saudi Arabia regarding VPT, with a focus on diagnostic tools, rubber dam isolation, and material selection. The aim was to identify practice variability and evidence-based gaps and propose strategies to standardise care.

**Methods and Materials:**

A cross-sectional study was conducted among 302 dental professionals using a validated online questionnaire. Data collection spanned December 2024 to early March 2025. Descriptive statistics, non-parametric tests, including the Mann–Whitney U and Kruskal–Wallis tests, were employed to compare practices across groups, while binary logistic regression identified predictors of good knowledge (≥70%). A P value < 0.05 was considered statistically significant.

**Results:**

Preoperative practices highlighted frequent use of pulp sensibility testing (79.1%) and periapical radiographs (50%), with cold testing as the preferred method (52.6%). However, advanced tools like CBCT were underutilised. Intraoperatively, 67.2% consistently used rubber dam isolation, while calcium hydroxide (22.5%) was the most commonly used pulp capping material, despite increasing adoption of mineral trioxide aggregate (MTA) and biodentine. Postoperatively, 46% adhered to a 3–6 month follow-up interval, relying on cold testing and percussion for assessment. Logistic regression revealed postgraduate education, specialisation, and frequency of VPT procedures as significant predictors of evidence-based practices.

**Conclusion:**

The findings highlight significant variability in VPT practices among dental professionals in Saudi Arabia, emphasising the need for targeted training programmes and standardised guidelines to bridge evidence-based gaps, improve clinical consistency, and optimise patient outcomes.

Vital pulp therapy (VPT) has emerged as an effective minimally invasive strategy aimed at preserving the vitality of the pulp in teeth affected by caries or trauma. This therapeutic approach includes treatments such as indirect pulp capping, direct pulp capping, partial pulpotomy, and full pulpotomy, each varying in the extent of pulp tissue management.^7,16^ The primary objective of VPT is to maintain the health and function of the dental pulp, thereby prolonging the lifespan of the natural tooth.^16,30^ Globally, VPT is viewed as a less invasive alternative to root canal treatment, offering numerous benefits, including the preservation of the tooth structure, reduced treatment costs, and improved long-term prognosis.^22^ The success of VPT is influenced by multiple factors, including accurate diagnosis of pulp status, proper case selection, strict adherence to aseptic protocols, the use of biocompatible materials, and adherence to evidence-based procedural protocols.^17,36^

Recent advancements in bioactive materials, such as mineral trioxide aggregate (MTA) and biodentine, have significantly improved clinical outcomes by promoting dentin bridge formation and pulp healing. Studies have reported high success rates for VPT procedures utilising these materials, even in cases with carious pulp exposure.^11^ Despite these advancements, variations in diagnostic and therapeutic approaches persist globally, reflecting differences in practitioner training, resource availability, and adherence to guidelines.^24^

In Saudi Arabia, the prevalence of dental caries and pulpitis remains a significant oral health concern, mirroring global trends and highlighting the need for effective management strategies such as VPT.^21^ Despite its potential, dental professionals in Saudi Arabia face challenges in implementing VPT, including variability in knowledge levels, inconsistent adherence to evidence-based guidelines, and limited integration of advanced diagnostic tools such as cone-beam computed tomography (CBCT).^4,12,35^ Additionally, traditional materials like calcium hydroxide continue to dominate clinical practice, despite the availability of superior bioactive alternatives like MTA and biodentine.^2,5^ These challenges suggest the need for a deeper understanding of current VPT practices among dental professionals to identify gaps and opportunities for improvement. While some studies in Saudi Arabia have explored specific aspects of vital pulp therapy (VPT), such as its application in primary teeth or its use among interns and paediatric dentists, there remains a notable gap in the literature.^12,13,34^

Comprehensive research examining the entire spectrum of clinical practices in VPT, covering preoperative, intraoperative, and postoperative phases, remains limited. This knowledge gap restricts a holistic understanding of how VPT is currently performed, particularly regarding the integration of diagnostic tools, material selection, procedural techniques, and follow-up protocols. Addressing this gap is essential to developing a clearer picture of current practices, identifying deficiencies, and generating actionable insights to standardise and improve VPT delivery. This study aimed to evaluate the clinical practices of dental professionals in Saudi Arabia across all phases of VPT, focusing on diagnostic methods, rubber dam usage, and material selection. By uncovering practice variations and evidence-based gaps, this research seeks to inform targeted interventions and policy recommendations that can improve clinical consistency, optimise patient outcomes, and strengthen pulp preservation strategies.

## METHODS AND MATERIALS

The study received ethical approval from the Research Ethics Committee of Jouf University (No: HAP-13-S001), Saudi Arabia. It was an observational study of a descriptive cross-sectional design. The present study was conducted among the registered dental professionals in Saudi Arabia, irrespective of their gender or area of practice. The study did not include those who refused to give their consent. Data collection commenced immediately after receiving the final ethics committee approval on 12 December 2024 and continued until early March 2025.

Due to logistical constraints, a non-probability snowball sampling technique was employed to collect data through an online Google Form. The principal investigator (PI) initially identified participants through professional and academic networks, including faculty mailing lists, dental association groups, and postgraduate alumni forums across Saudi Arabia. The survey link was distributed through platforms such as WhatsApp and email, and participants were encouraged to forward it to other eligible colleagues. To minimise duplication or network bias, the form was configured to accept only one response per Google account, and participants were required to confirm their email before submission. Respondents were informed about the study’s objectives and assured that their participation was entirely voluntary and anonymous. The inclusion criteria required participants to be actively practising dentistry and willing to provide consent, while the exclusion criteria excluded individuals not involved in dentistry or unwilling to consent. Confidentiality and anonymity were strictly maintained throughout the study, with no personally identifiable information included in the data processing or analysis. Based on a 95% confidence level and a ±6% margin of error for a national population of approximately 20,000 registered dentists, the estimated minimum sample size was 267. The final sample of 302 participants exceeded this requirement, ensuring acceptable precision for subgroup comparisons and reliable statistical analysis.

A structured questionnaire was developed with reference to previously published surveys on VPT practices and adapted to the Saudi context.^10^ Its content validity was reviewed by a panel of five endodontic experts, followed by a pilot test (n = 30), which led to minor refinements for clarity and comprehensibility. Cronbach’s α values for the preoperative, intraoperative, and postoperative domains were 0.81, 0.84, and 0.79, respectively, indicating good internal consistency.

The questionnaire was divided into three sections to comprehensively assess practitioners’ pre, intra, and postoperative knowledge and practices related to VPT. The first section focused on participant sociodemographics, capturing details such as gender, age, years of professional experience, postgraduate education, speciality, workplace, and frequency of VPT performed. The second section, addressing preoperative assessment, included nine questions exploring diagnostic tools (eg, pulp vitality tests, radiographic assessments), criteria for pulpitis classification, and decision-making processes. The third section covered intraoperative and postoperative practices, with ten questions on rubber dam usage, bleeding control methods, criteria for assessing pulp stump health after hemostasis (eg, visual inspection of colour, absence of bleeding, pulp texture, bleeding time), therapeutic approaches (eg, direct pulp capping, partial pulpotomy), and materials used for VPT, alongside two questions on follow-up intervals, clinical assessments, and the evaluation of treatment success rates. This structured approach ensured a comprehensive evaluation of VPT practices among practitioners, providing insights into current trends and areas for improvement.

The normality distribution of the study outcome variables (ie, preoperative, intraoperative, and postoperative assessments of VPT scores) was examined using the Kolmogorov-Smirnov and the Shapiro-Wilk tests for inferential statistics. We found that the scores were not normally distributed (P < 0.05), indicating the need for non-parametric tests. Hence, the statistical analyses performed in the present study included descriptive analysis, Mann–Whitney test, Kruskal–Wallis test, and binary logistic regression analysis. Responses were scored as 1 for each correct answer and 0 for incorrect or missing responses. Total knowledge and practice scores were calculated by summing across items, with higher scores reflecting greater adherence to evidence-based VPT practices. Because the distributions were non-normal, results are reported as median (IQR) instead of mean (SD). The descriptive analysis presented the median (IQR), frequencies, and percentages. For comparisons between two groups, the Mann–Whitney U test was used with effect size calculated as r = Z/√N. For comparisons across more than two groups, the Kruskal–Wallis H test was applied with η^2^ reported as the effect size. Where applicable, post-hoc pairwise comparisons were performed with Bonferroni adjustment to correct for multiple testing. This 70% threshold has been widely used in prior dental knowledge and competency assessments as a balanced indicator of adequate professional understanding, being neither overly lenient nor excessively strict.^33^

Multiple logistic regression analysis was conducted to determine significant predictors of total knowledge (≥70% vs < 70%). Adjusted odds ratios (AOR) with 95% confidence intervals were reported. In the present study, 40.4% of participants scored below 70%, while 59.6% achieved 70% or higher. We used this cutoff to categorise participants into groups with good knowledge and poor knowledge. Additionally, the 70% threshold is widely regarded by researchers as a practical and balanced division point, being neither overly lenient nor excessively strict. Data results were presented in tabular form, showing participant demographics, preoperative, intraoperative, and postoperative practices, as well as predictors of knowledge variability. All statistical tests were two-tailed, with a significance threshold set at P < 0.05. Statistical analyses were performed using SPSS version 27.

## RESULTS

Table 1 elaborates on the general characteristics of the study participants. A total of 302 respondents participated in the study, with slightly higher male participation. Most participants were 25–30 years old with 0–5 years of professional experience. The majority of participants obtained a postgraduate degree (58.3%), with only 18.9% being specialist endodontists. The least represented sector was the private practice, while most participants saw 5–10 patients per week. The frequency of VPT encounters varied, with only 15.9% of participants abstaining from it.

**Table 1:** General characteristics of the study participants

Variable	N	%
**Gender** Female Male	135 167	44.7 55.3
**Age** 25–30 36–40 >45	226 52 24	74.8 17.2 7.9
**Professional experience (years)** 0–5 6–10 11–15 16–20 ≥20	151 100 23 13 15	50.0 33.1 7.6 4.3 5.0
**Postgraduate degree** No Yes	123 179	40.7 59.3
**Speciality** None Dental public health Endodontics Maxillofacial surgery Operative dentistry Oral pathology Orthodontics Periodontics Prosthodontics	153 11 57 8 12 7 9 11 34	50.7 3.6 18.9 2.6 4.0 2.3 3.0 3.6 11.3
**If you answered to postgraduate degree ‘No’, please specify** Interns General dentistry	75 48	24.8 15.9
**For non-endodontists: Do you have any specialised training in endodontics?** *If you are an endodontist, please select ‘Not Applicable’.* Not applicable Workshops Basic training Advance training	147 10 129 16	48.7 3.3 42.7 5.3
**Workplace** Academic Government Private	125 132 45	41.4 43.7 14.9
**Number of patients seen per week** 5–10 10–15 15–20 20–25 25–30 30–40	185 55 34 16 10 2	61.3 18.2 11.3 5.3 3.3 0.7
**Continuous training** Yes No	79 223	26.2 73.8
**How often do you perform vital pulp therapies in your practice?** Daily Weekly Monthly Rarely None	50 85 50 69 48	16.6 28.1 16.6 22.8 15.9


Table 2 presents the preoperative assessment of VPT by dental professionals. A significant proportion of respondents (46.4%) selected pulp sensibility testing (eg, cold test, electric pulp tester) as the primary diagnostic tool for assessing the pulpal condition in deep carious lesions. Most respondents used periapical radiographs for preoperative radiographic evaluation, coupled with sensibility testing to assess the clinical status of the pulp. Regarding pulp sensibility tests, cold testing was most commonly used (52.6%), with Endo-Ice being the preferred agent for the cold test (48.0%). Meanwhile, most participants did not use electric pulp testing as their primary device (68.2%). For determining the extent of caries involvement and pulpal condition in deep carious lesions, a combination of radiographic and clinical findings was primarily relied upon (75.3%). To differentiate between reversible and irreversible pulpitis in deep carious lesions, 81.5% used pain intensity and duration combined with sensitivity. Lastly, prolonged pain after thermal stimulus was most commonly used to identify symptoms associated with irreversible pulpitis (30.8%).

**Table 2:** Preoperative assessment of vital pulp therapies

Variable	N	%
**What diagnostic tools do you typically use to assess the pulpal condition in deep carious lesions?** Clinical examination Clinical examination, pulp sensibility testing (eg, cold test, electric pulp tester) Clinical examination, radiographic assessment Pulp sensibility testing (eg, cold test, electric pulp tester) All of the above	21 140 13 42 86	7.0 46.4 4.3 13.9 28.5
**How do you evaluate the radiographic status of the tooth before treatment?** Bitewing radiograph Panoramic radiograph Periapical radiograph Periapical radiograph, bitewing radiograph Periapical radiograph, cone beam CT (CBCT) Periapical radiograph, cone beam CT (CBCT), bitewing radiograph Periapical radiograph, panoramic radiograph Periapical radiograph, panoramic radiograph, bitewing radiograph Periapical radiograph, panoramic radiograph, cone beam CT (CBCT)	9 3 151 69 6 3 30 26 5	3.0 1.0 50.0 22.8 2.0 1.0 9.9 8.6 1.7
**Do you routinely perform pulp sensibility testing before vital pulp therapy?** No Yes	63 239	20.9 79.1
**Which pulp sensibility tests do you commonly use?** Cold testing Cold testing, electric pulp testing Cold testing, electric pulp testing, heat testing Cold testing, heat testing Electric pulp testing Other	159 51 12 17 23 40	52.6 16.9 4.0 5.6 7.6 13.2
**Which agent do you prefer to use for cold test?** CO_2_ snow Endo-Frost Endo-Frost, CO_2_ snow Endo-Frost, Endo-Ice Endo-Frost, Endo-Ice, CO_2_ snow Endo-Frost, Endo-Ice, Ice stick Endo-Ice Endo-Ice, Ice stick Ice stick Other	16 45 3 24 6 3 145 10 5 45	5.3 14.9 1.0 7.9 2.0 1.0 48.0 3.3 1.7 14.9
**For electric pulp testing, what is your primary device?** Analytic technology pulp tester Digitest II pulp vitality tester I do not use electric pulp testing Parkell pulp vitality tester Vitality scanner No response	41 14 206 19 20 2	13.6 4.6 68.2 6.3 6.6 0.7
**How do you determine the extent of caries involvement and pulpal condition in deep carious lesions?** By probing the lesion with a dental explorer Clinical symptoms assessment Combination of radiographic and clinical findings Radiographic interpretation No answer	15 20 222 43 1	5.0 6.6 75.3 14.2 0.7
**What criteria do you use to differentiate reversible and irreversible pulpitis in deep carious lesions?** Extent of carious lesion and patient’s age Pain intensity and duration combined with sensitivity Presence of periapical radiolucency Response to local anaesthesia during treatment No answer	3 246 38 13 2	1.0 81.5 12.6 4.3 0.7
**What method do you use to identify the symptom frequently linked with irreversible pulpitis?** Pain that is worse at night Prolonged pain after thermal stimulus Prolonged pain after thermal stimulus, pain that is worse at night Prolonged pain after thermal stimulus, sharp pain that subsides quickly Prolonged pain after thermal stimulus, throbbing pain that radiates to the ear Sharp pain that subsides quickly after removal of stimulus Throbbing pain that radiates to the ear Throbbing pain that radiates to the ear, pain that is worse No response	12 93 42 29 60 47 13 4 2	4.0 30.8 13.9 9.6 19.9 15.6 4.3 1.3 0.7
		

Table 3 presents the study participants’ intraoperative assessment of VPT. The majority answered all of the above when asked about the factors influencing their decision-making process in selecting a therapeutic approach for VPT (43.4%). For the routine use of a rubber dam during the procedure, most participants answered ‘yes, always’ (67.2%). To control bleeding, applying pressure with a dry cotton pellet was most common (46.0%), followed by a NaOCl-soaked pellet (30.8%). The predominance of visual inspection of colour with the absence of further bleeding (49.7%) indicates reliance on simple chairside indicators, whereas magnification remained underused (71.2% reported no magnification). Pulpectomy was the preferred approach for managing cases of mechanical pulp exposure (61.9%). For managing deep carious lesions with evidence of irreversible pulpitis, pulp vitality status was the key approach (50.3%). When asked about the percentage of teeth treated with direct pulp capping that show successful outcomes, 32.1% reported a success rate of 51–75%. Finally, calcium hydroxide was the most commonly used material for VPT (22.5%).

**Table 3:** Intraoperative assessment of vital pulp therapies

Variable	N	%
**What factors influence your decision-making process when selecting a therapeutic approach for vital pulp therapy?** Ability to control the bleeding Pulpal status and vitality Pulpal status and vitality, ability to control the bleeding Pulpal status and vitality, rubber dam application Pulpal status and vitality, rubber dam application, ability Pulpal status and vitality, size and depth of the carious lesion Rubber dam application Rubber dam application, ability to control the bleeding Size and depth of the carious lesion Size and depth of the carious lesion, rubber dam application No answer All of the above	12 22 4 6 25 74 3 10 9 4 2 131	4.0 7.3 1.3 2.0 8.3 24.5 1.0 3.3 3.0 1.3 0.7 43.4
**Do you routinely use a rubber dam during vital pulp therapy?** No Yes, always Yes, sometimes No answer	61 203 36 2	20.2 67.2 11.9 0.7
**How do you control bleeding during the procedure?** Controlling bleeding by applying pressure with cotton pellet soaked with chlorohexidine Controlling bleeding by applying pressure with cotton pellet soaked with local anaesthesia Controlling bleeding by applying pressure with cotton pellet soaked with NaOCl Controlling bleeding by applying pressure with dry cotton pellet No response	3 65 93 139 2	1.0 21.5 30.8 46.0 0.7
**Do you use magnification during vital pulp therapy?** Loupes (please specify magnification level below) 2.5× 3.0× 3.5× Microscope No magnification No answer	26 7 32 17 215 5	8.6 2.3 10.6 5.6 71.2 1.6
**How do you assess the health of the pulp stump after controlling bleeding?** Visual inspection of colour, absence of further bleeding Visual inspection of colour, pulp texture upon probing Absence of further bleeding, pulp texture upon probing No answer	150 70 60 22	49.7 23.219.9 7.3
**How do you manage cases with mechanical exposure of the pulp?** Direct pulp capping Extraction Full pulpotomy Partial pulpotomy Pulpectomy No answer	12 26 51 24 187 2	4.0 8.6 16.9 7.9 61.9 0.7
**How do you manage deep carious lesions with evidence of irreversible pulpitis?** Age of the patient Patient’s dental history Pulp vitality status Size of the carious lesion No answer	80 52 152 16 2	26.5 17.2 50.3 5.3 0.7
**What percentage of teeth treated with direct pulp capping in your practice show successful outcomes?** 25–50% 51–75% Less than 25% More than 75% No answer	75 97 51 77 2	24.8 32.1 16.9 25.5 0.7
**What material do you commonly used for vital pulp therapy?** All of the above Biodentine Biodentine, bio-aggregate, calcium hydroxide Calcium hydroxide MTA MTA, biodentine MTA, biodentine, bio-aggregate, calcium hydroxide MTA, biodentine, calcium hydroxide MTA, biodentine, root repair material (RRM) putty MTA, biodentine, root repair material (RRM) putty, bio-aggregate MTA, biodentine, root repair material (RRM) putty, calcium hydroxide MTA, calcium hydroxide MTA, root repair material (RRM) putty MTA, root repair material (RRM) putty, Calcium hydroxide Root repair material (RRM) putty No answer	29 6 2 68 54 2 3 27 5 5 8 64 12 9 6 2	9.6 2.0 0.7 22.5 17.9 0.7 1.0 8.9 1.7 1.7 2.6 21.2 4.0 3.0 2.0 0.7
Abbreviations: MTA – mineral trioxide aggregate; RRM – root repair; NaOCl – sodium hypochlorite

Table 4 presents the study participants’ postoperative assessment of VPT. The majority answered 3-6 months when asked about the factors influencing their decision-making process in selecting the typical duration of follow-up after VPT (46.0%). For the clinical assessments performed during follow-up, most participants answered cold test, tenderness to percussion and/or palpation (30.1%).

**Table 4:** Postoperative assessment of VPT

Variable	N	%
**What is the typical duration of follow-up after vital pulp therapy?** 1 month 1 week 1 year 3–6 months No answer	112 39 10 139 2	37.1 12.9 3.3 46.0 0.7
**What clinical assessments do you perform during follow-up?** Adjustment of occlusion Cold test Cold test, adjustment of occlusion Cold test, electric pulp test Cold test, electric pulp test, tenderness to percussion and/or palpation Cold test, tenderness to percussion and/or palpation Cold test, tenderness to percussion and/or palpation, adjust of occlusion Electric pulp test Electric pulp test, tenderness to percussion and/or palpation Tenderness to percussion and/or palpation Tenderness to percussion and/or palpation, adjustment of occlusion No answer	4 38 4 38 33 91 11 11 4 58 10 2	1.3 12.6 1.3 12.6 10.9 30.1 3.6 3.6 1.3 19.2 3.3 0.7


Table 5 presents the median (IQR) knowledge scores in the preoperative assessment of VPT across participant characteristics. Significant differences were observed for postgraduate degree (U = 9872, P = 0.012, r = 0.18), specialty (H =16.927, P = 0.004, η^2^ = 0.05), and continuous training (U = 9564, P = 0.006, r = 0.20). Practitioners with postgraduate qualifications, those in endodontics or operative dentistry, and those engaged in continuous training demonstrated higher knowledge scores. Other variables, including gender, age, experience, workplace, patients per week, and frequency of VPT, were not significantly associated with knowledge scores. These findings suggest that educational background, clinical specialisation, and ongoing training are important determinants of preoperative knowledge in VPT.

**Table 5:** Knowledge scores by participant characteristics

Variable	Median (IQR) by group	Test statistic	P value	Effect size
Gender	Male: 12 (10–14); Female: 12 (10–13)	U = 11215	0.187	r = 0.07
Age (years)	25–35: 12 (10–14); 36–45: 12 (10–13); > 45: 11 (9–13)	H = 5.832	0.212	η^2^ = 0.01
Professional experience (years)	0–5: 12 (10–13); 6–10: 12 (10–14); 11–15: 12 (10–14); 16–20: 11 (9–13); > 20: 11 (9–12)	H = 7.364	0.118	η^2^ = 0.02
Postgraduate degree	Yes: 13 (11–15); No: 11 (9–13)	U = 9872	0.012	r = 0.18
Speciality (PG only)	Endodontics: 13 (11–15); Operative dentistry: 13 (11–14); Periodontics: 12 (10–14); Prosthodontics: 12 (10–13); Orthodontics: 11 (9–13); Oral pathology: 11 (9–12); None: 11 (9–12)	H = 16.927	0.004	η^2^ = 0.05
Non-PG subgroup	Interns: 12 (10–13); General dentistry: 12 (10–14); Not applicable: 11 (9–12)	H = 4.072	0.281	η^2^ = 0.01
Workplace	Academic: 12 (10–14); Private: 12 (10–13); Government: 12 (10–14)	H = 5.083	0.165	η^2^ = 0.01
Patients per week	5–10: 11 (9–12); 11–15: 12 (10–14); 16–20: 12 (10–14); 21–25: 12 (10–14); > 25: 13 (11–15)	H = 6.941	0.142	η^2^ = 0.02
Frequency of VPT	Rarely: 12 (10–13); Monthly: 12 (10–14); Weekly: 12 (10–14); Daily: 13 (11–15); None: 11 (9–12)	H = 8.326	0.076	η^2^ = 0.02
Continuous training	Yes: 13 (11–15); No: 11 (9–13)	U = 9564	0.006	r = 0.20
Post-hoc analysis for speciality: endodontics vs none (P = 0.004), operative dentistry vs none (P = 0.012), endodontics vs orthodontics (P = 0.019). Post-hoc analysis for postgraduate degree: postgraduates vs non-postgraduates (P = 0.012). Post-hoc analysis for continuous training: participants with ongoing training vs those without (P = 0.006). (Post-hoc tests were conducted only when the omnibus test was significant).

Table 6 presents the median (IQR) intraoperative practice scores of VPT across participant characteristics. Significant differences were observed for age (H = 12.412, P = 0.015, η^2^ = 0.04), specialty (H = 18.635, P = 0.002, η^2^ = 0.06), and patients per week (H = 14.829, P = 0.005, η^2^ = 0.05). Younger practitioners and those treating a higher weekly patient load showed higher intraoperative practice scores. Endodontists and operative dentists reported higher scores than colleagues from surgical or non-clinical specialities. No significant differences were observed for gender, professional experience, postgraduate status, non-PG subgroup, workplace, frequency of VPT, or continuous training (all P > 0.05). These findings suggest that intraoperative practice behaviours are influenced primarily by age, clinical speciality, and workload.

**Table 6:** Practice scores by participant characteristics

Variable	Median (IQR) by group	Test statistic	P value	Effect size
Gender	Male: 12 (10–14); Female: 12 (10–13)	U = 11327	0.812	r = 0.02
Age (years)	25–35: 13 (11–15); 36–45: 12 (10–14); > 45: 11 (9–13)	H = 12.412	**0.015**	η^2^ = 0.04
Professional experience (years)	0–5: 12 (10–13); 6–10: 12 (10–14); 11–15: 12 (10–14); 16–20: 11 (10–13); > 20: 11 (9–13)	H = 5.921	0.205	η^2^ = 0.01
Postgraduate degree	Yes: 12 (10–14); No: 12 (10–13)	U = 10652	0.143	r = 0.07
Specialty (PG only)	Endodontics: 13 (11–15); Operative dentistry: 13 (11–15); Periodontics: 12 (10–14); Prosthodontics: 12 (10–13); Orthodontics: 11 (9–13); Oral pathology: 11 (9–12); None: 11 (9–12)	H = 18.635	**0.002**	η^2^ = 0.06
Non-PG subgroup	Interns: 12 (10–13); General dentistry: 12 (10–14); Not applicable: 11 (9–12)	H = 3.578	0.310	η^2^ = 0.01
Workplace	Academic: 12 (10–14); Private: 12 (10–14); Government: 12 (10–13)	H = 2.844	0.241	η^2^ = 0.01
Patients per week	5–10: 11 (9–13); 11–15: 12 (10–13); 16–20: 13 (11–15); 21–25: 13 (11–15); > 25: 13 (11–15)	H = 14.829	**0.005**	η^2^ = 0.05
Frequency of VPT	Rarely: 12 (10–14); Monthly: 12 (10–14); Weekly: 12 (10–13); Daily: 12 (10–14); None: 11 (9–12)	H = 4.067	0.385	η^2^ = 0.01
Continuous training	Yes: 12 (10–14); No: 12 (10–13)	U = 9874	0.189	r = 0.06
Post-hoc analysis for age: 25–35 vs > 45 years (P = 0.021). Post-hoc analysis for speciality: endodontics vs none (P = 0.004), operative dentistry vs none (P = 0.012). Post hoc analysis for patient load: 5–10 vs 16–20 per week (P = 0.009), 5–10 vs 21–25 (P = 0.005). (Post-hoc tests were conducted only when the omnibus test was significant).

Table 7 presents the median (IQR) postoperative assessment scores of VPT across participant characteristics. Omnibus tests showed significant differences for age (H = 35.220, P < 0.001, η^2^ = 0.09), professional experience (H = 24.033, P < 0.001, η^2^ = 0.07), specialty (H = 54.264, P < 0.001, η^2^ = 0.15), non-postgraduate subgroup (H = 9.340, P = 0.009, η^2^ = 0.02), endodontic training among non-endodontists (H = 20.930, P < 0.001, η^2^ = 0.06), patients per week (H = 26.437, P < 0.001, η^2^ = 0.07), and frequency of VPT (H = 79.014, P < 0.001, η^2^ = 0.25). In contrast, no significant differences were found for gender (U = 12080, P = 0.214, r = 0.06), postgraduate degree (H = 4.820, P = 0.090, η^2^ = 0.01), workplace (H = 0.789, P = 0.852, η^2^ = 0.00), or continuous training (U = 8610, P = 0.730, r = 0.02). These findings indicate that postoperative practices varied significantly with clinician demographics, speciality, training subgroup, and workload, while no meaningful differences were observed for gender, degree status, or workplace.

**Table 7:** Postoperative scores by participant characteristics

Variable	Median (IQR) by group	Test statistic	P value	Effect size
Gender	Male: 1 (1–2); Female: 1 (1–2)	U = 12080	0.214	r = 0.06
Age (years)	25–30: 1 (1–2); 31–35: 2 (1–2); 36–40: 2 (2–2); 41–45: 1 (1–2); 46–50: 1 (1–2)	H = 35.220	< 0.001	η^2^ = 0.09
Experience (years)	20 or more: 1 (1–1); 6-10: 2 (1–2); 0-5: 1 (1–2); 16 – 20: 1 (1–2); 11-15: 1 (1–2)	H = 24.033	< 0.001	η^2^ = 0.07
Postgraduate degree	Yes: 1 (1–2); No: 1 (1–2); Yes, No: 1 (1–1)	H = 4.820	0.090	η^2^ = 0.01
Specialty (PG only)	Orthodontics: 1 (1–1); Endodontics: 2 (1–2); Operative Dentistry: 1 (1–2); Periodontics: 2 (2–2); Not applicable: 1 (1–2); Prosthodontics: 2 (1–2); Maxillofacial Surgery: 1 (1–1); Dental public health: 1 (1–1); Oral pathology: 2 (1–2)	H = 54.264	< 0.001	η^2^ = 0.15
Non‑PG subgroup	Not applicable: 2 (1–2); Interns: 1 (1–2); General Dentistry: 1 (1–2)	H = 9.340	0.009	η^2^ = 0.02
Endodontic training (non‑endodontists)	No, no specialised training: 1 (1–1); Not applicable: 2 (1–2); Yes, basic training: 1 (1–2); Yes, advanced training: 1 (1–2); No, but have attended workshops: 1 (1–2)	H = 20.930	< 0.001	η^2^ = 0.06
Workplace (primary)	Academic institution: 1 (1–2); Private practice: 1 (1–2); Government Hospital: 1 (1–2); Government/private both: 1 (1–2)	H = 0.789	0.852	η^2^ = 0.00
Patients per week	05 – 10: 1 (1–2); 10 – 15: 1 (1–2); 15 – 20: 2 (1–2); 30 – 40: 2 (2–2); 25 – 30: 2 (1–2); 20 – 25: 2 (2–2)	H = 26.437	< 0.001	η^2^ = 0.07
Continuous training	No: 1 (1–2); Yes: 1 (1–2)	U = 8610	0.730	r = 0.02
Frequency of VPT	None: 1 (1–2); Rarely: 1 (1–1); Weekly: 2 (1–2); Monthly: 1 (1–1); Daily: 2 (2–2)	H = 79.014	< 0.001	η^2^ = 0.25
Post-hoc analysis for age: 25–35 vs > 45 years (P = 0.035), 36–45 vs > 45 years (P = 0.012). Post-hoc analysis for professional experience: 11–15 vs > 20 years (P = 0.037), 6–10 vs > 20 years (P = 0.002). Post-hoc analysis for specialty: orthodontics vs endodontics (P = 0.007), orthodontics vs periodontics (P = 0.009), maxillofacial vs periodontics (P = 0.039), dental public health vs endodontics (P = 0.009), dental public health vs periodontics (P = 0.013), no specialty vs endodontics (P = 0.001). Post-hoc analysis for postgraduate status: not applicable vs interns (P = 0.013). Post-hoc analysis for patient load: 5–10 vs 16–20 per week (P = 0.001), 5–10 vs 21–25 (P = 0.002), 11–15 vs 21–25 (P = 0.031). Post-hoc analysis for frequency of VPT: rarely vs daily (P < 0.001), daily vs monthly (P < 0.001), daily vs weekly (P < 0.001), none vs daily (P < 0.001).

Table 8 presents the significant predictors of total knowledge of preoperative, intraoperative, and postoperative assessments of VPT. The results are expressed as adjusted odds ratios (AORs) with corresponding 95% confidence intervals (CIs). The results indicated that professional experience, postgraduate degree, speciality, workplace, number of patients seen per week, and frequency of performing VPT were significant predictors of total knowledge scores. Dental professionals with 6–10 years of experience were 73% less likely to achieve a score of 70% or above compared to those with 0–5 years of experience (AOR = 0.27, P = 0.008). Similarly, those with 11–15 years of experience were 86% less likely (AOR = 0.14, P = 0.015), 16–20 years were 88% less likely (AOR = 0.12, P = 0.026), and more than 20 years of experience were 96% less likely (AOR = 0.04, P = 0.003) to have good knowledge compared to the 0-5 years group. Dental professionals with a postgraduate degree were 34.34 times more likely to achieve a score of 70% or above compared to those without postgraduate education (AOR = 34.34, P < 0.001). Regarding speciality, participants in dental public health (AOR = 0.03, P = 0.004) and maxillofacial surgery (AOR = 0.04, P = 0.009) were significantly less likely to achieve high knowledge scores compared to general dentists without a speciality. Endodontists, despite showing an adjusted odds ratio of 0.20 (P = 0.036), are clinically more likely to possess higher knowledge in VPT due to their specialised training and frequent involvement in such procedures. This lower odds ratio may reflect the influence of other adjusted factors, including postgraduate education and workplace setting. Prosthodontists were 6.3 times more likely to achieve high knowledge scores compared to the reference group (AOR = 6.30, P = 0.049). Those working in government institutions were 88% less likely to achieve a score of 70% or above compared to those working in academia (AOR = 0.12, P < 0.001). Participants seeing 11–15 patients per week were 80% less likely to score ≥70% compared to those seeing 5–10 patients weekly (AOR = 0.20, P = 0.011). Finally, those who performed vital pulp therapies monthly were 82% less likely to achieve a score of 70% or above compared to participants who never performed VPT (AOR = 0.18, P = 0.024).

**Table 8:** Factors associated with total knowledge of preoperative, intraoperative, and postoperative assessment of vital pulp therapies (< 70% vs ≥ 70%)

Variable	AOR (95% confidence interval)	P value
**Professional experience (years)** 0–5 6–10 11–15 16–20 ≥20	1 0.27 (0.10, 0.71) 0.14 (0.03, 0.68) 0.12 (0.02, 0.78) 0.04 (0.01, 0.34)	0.008 0.015 0.026 0.003
**Postgraduate degree** No Yes	1 34.34 (9.21, 127.96)	< 0.001
**Speciality** Not applicable Dental public health Endodontics Maxillofacial surgery Operative dentistry Oral pathology Orthodontics Periodontics Prosthodontics	1 0.03 (0.01, 0.31) 0.20 (0.04, 0.90) 0.04 (0.01, 0.45) – 1.34 (0.07, 26.14) 0.01 (0.01, 0.19) – 6.30 (1.01, 39.39)	0.004 0.036 0.009 – 0.846 0.001 – 0.049
**Workplace** Academic Government Private	1 0.12 (0.05, 0.27) 0.44 (0.14, 1.40)	< 0.001 0.164
**Number of patients seen per week** 5–10 11–15 16–20 22–25 26–30 31–40	1 0.20 (0.06, 0.69) 3.14 (0.94, 10.50) 1.56 (0.19, 12.79) 0.17 (0.02, 1.44) –	0.011 0.063 0.678 0.104 –
**How often do you perform vital pulp therapies in your practice?** None Rarely Monthly Weekly Daily	1 3.16 (0.82, 12.16) 0.18 (0.04, 0.80) 1.17 (0.31, 4.39) 2.45 (0.48, 12.50)	0.095 0.024 0.813 0.283


**Fig 1:**
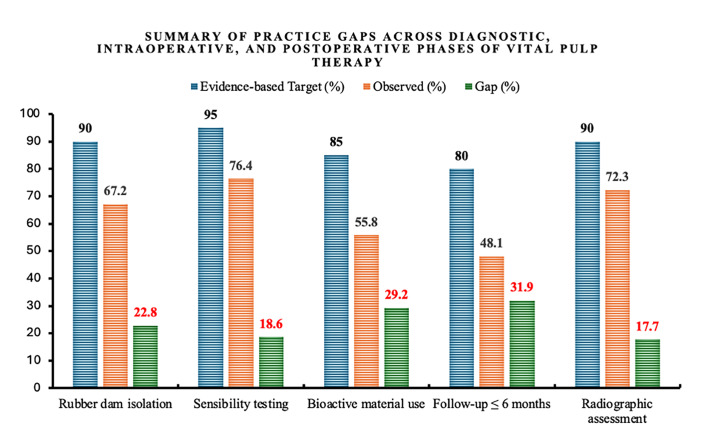
Summary of practice gaps across diagnostic, intraoperative, and postoperative phases of vital pulp therapy. Comparison of observed clinical behaviours (orange bars) with evidence-based guideline targets (blue bars) and corresponding practice gaps (green bars) across diagnostic, intraoperative, and postoperative phases. Evidence-based targets were extrapolated from qualitative recommendations in the ESE (2019) and AAE (2021) position statements and converted into approximate quantitative benchmarks (80-95%) to visualise compliance gaps, following accepted benchmarking methods.^16^ The largest gaps were noted in timely follow-up (31.9%) and bioactive material use (29.2%).

A consolidated comparison of observed clinical behaviours with evidence-based guideline targets is presented in Figure 1, showing the main practice gaps across diagnostic, intraoperative, and postoperative phases of VPT. The largest deficiencies were observed in timely follow-up (gap = 31.9 %) and bioactive material use (gap = 29.2 %). Evidence-based benchmark adherence levels were extrapolated from qualitative recommendations outlined in the European Society of Endodontology (2019) and American Association of Endodontists (2021) position statements and expressed as approximate quantitative targets (80–95 %) for comparative visualisation.^1,14^

## DISCUSSION

This study examined diagnostic, intraoperative, and follow-up practices in VPT among dental professionals in Saudi Arabia. Three central observations emerged: (1) considerable variability in diagnostic protocols and limited use of advanced tools; (2) inconsistent adherence to evidence-based intraoperative practices, particularly in isolation and material selection; and (3) lack of uniformity in follow-up intervals across clinicians. These findings underscore the persistent gap between clinical routine and contemporary evidence-based recommendations.

A summarised depiction of these discrepancies is illustrated in Figure 1, which presents benchmarked comparisons between observed practices and qualitative guideline-derived evidence-based targets (converted into approximate quantitative thresholds for visual comparison).^1,14,16^

Preoperative practices are fundamental to the success of VPT, as accurate diagnosis forms the basis for the appropriate treatment decisions. The study showed that pulp sensibility testing (79.1%) and cold testing (52.6%) are frequently used as primary diagnostic methods, reflecting a positive trend toward objective evaluation (Table 2). These methods offer valuable insights into pulpal vitality, helping clinicians differentiate between reversible and irreversible pulpitis and guide their treatment choices.^29^ The use of periapical radiographs (50.0%) as a routine diagnostic aid also reinforces their significance in assessing periapical status and the extent of pulpal involvement. However, the infrequent utilisation of advanced imaging tools such as CBCT points to a missed opportunity to enhance diagnostic precision, particularly in cases involving deep caries or trauma. As shown in Figure 1, diagnostic practices remain below recommended benchmarks, emphasising the need for improved access and training in advanced imaging modalities. CBCT enables three-dimensional visualisation of the pulp and surrounding structures, significantly enhancing treatment planning and outcomes.^26^ Additionally, the continued dependence on subjective indicators such as pain intensity and duration (81.5%) highlights the importance of more objective diagnostic frameworks. In this analysis, preoperative knowledge scores differed significantly by postgraduate qualification, speciality, and continuing education (Table 5), suggesting that advanced academic training and ongoing professional development contribute to greater diagnostic accuracy. Structured education modules and national guidelines promoting the routine use of objective tools could help narrow these diagnostic gaps.^9,21,30^

Intraoperative practices demonstrated a generally positive trend, with 67.2% of respondents consistently employing rubber dam isolation (Table 3). The use of rubber dam isolation is essential for maintaining asepsis, preventing microbial contamination, and improving restoration longevity. This finding aligns with prior research showing that rubber dam usage enhances procedural reliability and reduces postoperative infection risk.^15,23^ Nevertheless, the 32.8% of practitioners who do not consistently employ it reveal an area for targeted improvement, particularly among general practitioners citing time constraints or insufficient training. Incorporating rubber dam application into undergraduate and continuing education programmes may help strengthen this practice.^25^ Calcium hydroxide remained the most frequently used material for direct pulp capping (22.5%), followed by MTA and biodentine (Table 3). Although calcium hydroxide is cost-effective and historically prevalent, its long-term clinical limitations have been documented. The increasing adoption of bioactive materials such as MTA and biodentine is encouraging and suggests a gradual move toward evidence-based care. These materials demonstrate superior sealing ability and biocompatibility, promoting dentin regeneration.^11,27^ As visualised in Figure 1, bioactive material usage falls roughly 30% short of evidence-based targets, underscoring the need for institutional initiatives and clinical training programmes to encourage wider adoption.

The finding that 46.0% of practitioners controlled bleeding using a dry cotton pellet and 30.8% with NaOCl-soaked pellets illustrates continued reliance on conventional hemostatic methods (Table 3). Although these methods can be effective, the underuse of advanced hemostatic agents and magnification aids highlights an opportunity for enhancement. Incorporating such technologies into standard practice can enhance precision and treatment outcomes. Hemostatic agents, such as ferric sulfate and calcium chloride, have been shown to effectively control bleeding while maintaining pulp vitality and creating a conducive environment for the application of bioactive materials, MTA, and biodentine.^20^ In assessing pulp stump health after bleeding control, most practitioners reported combining visual inspection of pulp colour with the absence of further bleeding, while smaller groups considered combinations that included pulp texture upon probing. This reliance on simple chairside indicators reflects common clinical teaching, though it suggests that more nuanced assessment methods are underutilised. Similarly, the use of magnification tools, such as dental loupes or operating microscopes, improves visualisation, enhances precision, and allows for more accurate removal of carious tissue and assessment of pulp vitality.^8^ These technologies can significantly reduce procedural errors and improve treatment outcomes, particularly in cases involving deep caries or mechanical pulp exposure. The relatively limited adoption of magnification tools and bioactive materials observed in this study may be influenced by practical barriers, including material cost, limited access to equipment, and insufficient clinical training during both undergraduate education and professional practice. These constraints, also reported in regional studies, highlight the need for targeted workshops, institutional support, and continuing education programmes to promote the broader integration of evidence-based VPT practices.^3,19^

In the current analysis, intraoperative performance scores showed significant variation across age, speciality, and patient load (Table 6). Younger clinicians, endodontists, and those treating more patients weekly demonstrated higher adherence to evidence-based practices. This suggests that clinical experience, workload, and specialisation meaningfully influence practice consistency.

Postoperative follow-up practices were moderately encouraging, with 46% of practitioners adhering to 3–6-month recall intervals (Table 4). This rate exceeds some previous reports, though it still lags behind international expectations. The European Society of Endodontology recommends 6-month and 12-month evaluations following VPT to assess healing and vitality.^14^ As highlighted in Figure 1, follow-up adherence remains the widest practice gap (31.9%), signalling a need for clearer national and institutional recall protocols. This variation underscores the absence of standardised guidelines for follow-up intervals in VPT, highlighting the need for consensus to optimise patient care.^22^ The use of cold testing and tenderness to percussion as primary evaluation methods demonstrates the reliance on practical and accessible tools for assessing pulp vitality (Table 4). The integration of advanced diagnostic tools, such as CBCT or laser Doppler flowmetry, during follow-ups could provide more precise evaluations of pulp status and periapical healing. Such comprehensive monitoring protocols would not only enhance diagnostic accuracy but also improve the overall success rates of VPT, reinforcing its role as a minimally invasive alternative to root canal therapy.^21^

The findings of this study revealed that 40.4% of participants scored below 70% on the knowledge assessment, categorising them as having poor knowledge, while 59.6% achieved 70% or higher, demonstrating good knowledge. This threshold served as a balanced criterion for identifying gaps in knowledge and practice among dental practitioners in Saudi Arabia. The regression analysis identified postgraduate education (AOR = 34.34; P < 0.001), speciality, and frequency of VPT procedures performed as significant independent predictors of achieving good knowledge (≥ 70%). Practitioners with postgraduate education were significantly more likely to score above the 70% threshold, reflecting the importance of advanced training in fostering evidence-based practices. These individuals demonstrated a higher likelihood of incorporating modern diagnostic tools, such as CBCT, and bioactive materials like MTA and biodentine into their clinical workflows.^5,21,27^ Similarly, specialists, particularly those in operative dentistry and endodontics, showed greater adherence to standardised protocols, such as the routine use of rubber dam isolation and advanced therapeutic techniques. In contrast, general practitioners predominantly relied on traditional methods, such as the use of calcium hydroxide, which underscores the disparity in training and resource utilisation between generalists and specialists. The frequency of VPT procedures performed was another key predictor, with practitioners who regularly performed VPT demonstrating higher competency and confidence in their clinical approach. Regular exposure to VPT was associated with better integration of contemporary techniques and materials, emphasising the role of clinical experience in refining skills and staying aligned with evolving best practices.^32^ These findings highlight critical areas for intervention. Enhancing postgraduate and speciality training, coupled with structured clinical rotations, can bridge knowledge gaps and standardise VPT practices. Additionally, implementing continuing education programmes focused on the adoption of advanced diagnostic tools and bioactive materials can ensure consistent and evidence-based care. Such initiatives are essential for improving patient outcomes and elevating the overall quality of VPT practices in Saudi Arabia.

The findings of this study align with existing research in several aspects while also highlighting advancements and persistent challenges in VPT practices. Similar to earlier studies conducted in Saudi Arabia and globally, limited use of advanced diagnostic tools like CBCT was observed, with practitioners primarily relying on periapical radiographs and clinical examinations. This underscores ongoing barriers to adopting advanced technologies despite their potential to improve diagnostic precision.^6,12,22^

In terms of rubber dam usage, the results reflect significant variability, often attributed to perceived time constraints or insufficient training. While 67.2% of practitioners in this study consistently use rubber dams, a substantial proportion still neglect this evidence-based practice, indicating the need for targeted training programmes to improve adherence.^2,25^

Regarding material preferences, the continued reliance on calcium hydroxide mirrors trends identified in previous research, where its use was linked to familiarity and cost. However, the growing adoption of bioactive materials like MTA and biodentine in this study suggests a gradual shift towards more evidence-based practices.^12^ Additionally, 46% of practitioners adhered to 3–6 month follow-up intervals, demonstrating a proactive approach compared to the broader 6–12 month range often reported. This variation in follow-up intervals highlights the need for standardised guidelines to enhance consistency and optimise patient care in VPT.^14^ This lack of standardised guidelines for follow-up intervals underscores the need for consensus to enhance consistency and optimise patient care in VPT.

Consistent with prior research, this study also identifies gaps between practitioners’ knowledge and adherence to evidence-based protocols, evident in areas such as rubber dam usage, diagnostic tool adoption, and material preferences.^35^ What sets this study apart is its comprehensive scope, evaluating the full continuum of VPT from preoperative assessments to intraoperative decision-making and postoperative follow-up. Unlike previous cross-sectional studies that primarily focused on isolated aspects or general awareness, our research provides a detailed analysis of clinical decision-making processes and procedural variations among licensed dental professionals in Saudi Arabia. This broader and deeper approach offers clearer insights into the specific phases where these gaps occur, enabling the development of more precise, evidence-based recommendations to improve clinical outcomes and advance standardised VPT practices.

The study offers valuable insights into VPT practices in Saudi Arabia, but several limitations must be considered. The reliance on self-administered questionnaires introduces the potential for reporting bias, as responses are self-reported and unverifiable, potentially leading to overestimation of adherence to evidence-based practices. The use of non-probability sampling further limits the representativeness of the sample, restricting the generalizability of the findings to the broader dental community. Additionally, the cross-sectional design captures practices at a single point in time, failing to account for evolving trends or longitudinal variations in clinical behaviour. The regional focus of the study limits the applicability of findings to other contexts with different healthcare systems and resources. Finally, the study’s exclusion of patient outcomes means it evaluates practitioners’ knowledge and practices without assessing their direct impact on clinical success, which would provide a more comprehensive understanding of VPT efficacy. These limitations underscore the need for future research incorporating broader sampling, longitudinal designs, and clinical outcome assessments.

Despite these limitations, the findings have significant implications for clinical practice in Saudi Arabia. The widespread use of basic diagnostic tools, traditional materials like calcium hydroxide, and inconsistent application of rubber dams highlight critical areas for improvement. Addressing these gaps through the development and dissemination of standardised guidelines can ensure more consistent and effective VPT practices.

For instance, increasing the adoption of advanced diagnostic tools like CBCT and promoting the use of bioactive materials such as MTA and biodentine could significantly enhance treatment outcomes. Furthermore, targeted educational programmes emphasising the importance of rubber dam isolation and advanced intraoperative techniques can help bridge the knowledge gap among practitioners. Future research should prioritise developing evidence-based guidelines, evaluating the impact of targeted training programmes, and addressing systemic barriers to adopting advanced tools in VPT. Additionally, incorporating patient-centred outcomes will provide a comprehensive understanding of VPT’s efficacy and its impact on oral health.

## CONCLUSION

This cross-sectional survey provides a comprehensive assessment of prevailing VPT practices in Saudi Arabia. The findings demonstrate notable variability across diagnostic, intraoperative, and postoperative phases, reflecting both progress and persisting gaps in clinical adoption of evidence-based approaches. Key areas requiring attention include the consistent use of rubber dam isolation, wider integration of bioactive materials such as MTA and biodentine, and the establishment of structured follow-up protocols to ensure long-term treatment success. Differences in practice associated with postgraduate education, speciality, and clinical exposure highlight the importance of continuing education and hands-on training. Strengthening these areas through institutional support and national-level clinical guidelines can help harmonise VPT practices and promote more standardised, evidence-based patient care across the Kingdom.

### Acknowledgement

This work was funded by the Deanship of Graduate Studies and Scientific Research at Jouf University under grant no. (DGSSR-2024-01-01166).
